# Supplementary material for: A high-resolution mRNA expression time course of embryonic development in zebrafish
Source: eLife. 2017 Nov 16;6:e30860. doi: 10.7554/eLife.30860 (PMC5690287; doi:10.7554/eLife.30860)
Supplement: Supplementary file 6. [file elife-30860-supp6.zip › biolayout-clusters-files/Cluster197-genes.html]

Cluster197


# Cluster197: Genes

| Ensembl ID | Gene Name | Chr | Start | End | Biotype |
| --- | --- | --- | --- | --- | --- |
| ENSDARG00000021370 | actr6 | 25 | 19487367 | 19496141 | protein\_coding |
| ENSDARG00000009864 | ddx55 | 10 | 3397567 | 3415954 | protein\_coding |
| ENSDARG00000057105 | nle1 | 10 | 23101289 | 23130403 | protein\_coding |
| ENSDARG00000044402 | nop16 | 21 | 36927259 | 36934481 | protein\_coding |
| ENSDARG00000091124 | rps19bp1 | 6 | 247022 | 249989 | protein\_coding |
| ENSDARG00000007092 | xab2 | 3 | 53062377 | 53095884 | protein\_coding |
| ENSDARG00000044102 | znf593 | 17 | 24666377 | 24668415 | protein\_coding |
